# Household contact non-attendance of clinical evaluation for tuberculosis: a pilot study in a high burden district in South Africa

**DOI:** 10.1186/s12879-018-3010-3

**Published:** 2018-03-05

**Authors:** Gladys Kigozi, Michelle Engelbrecht, Christo Heunis, André Janse van Rensburg

**Affiliations:** Centre for Health Systems Research & Development, University of the Free State, P. O. Box 339, Bloemfontein, 9300 South Africa

**Keywords:** Household contact investigation, Tuberculosis, Active case finding, Clinical evaluation, Free State Province

## Abstract

**Background:**

In 2012, the World Health Organization launched guidelines for systematically investigating contacts of persons with infectious tuberculosis (TB) in low- and middle-income countries. As such, it is necessary to understand factors that would influence successful scale-up. This study targeted household contacts of newly-diagnosed infectious TB patients in the Mangaung Metropolitan district to explore factors associated with non-attendance of clinical evaluation.

**Method:**

In September–October 2016, a pilot study of household contacts was conducted. At each of the 40 primary health care (PHC) facilities in the district, at least one out of four types of TB index cases were purposefully selected. These included children <5 years, smear-positive cases, HIV co-infected cases, and multidrug-resistant TB (MDR-TB) cases. Trained fieldworkers administered questionnaires and screened contacts for TB symptoms. Those with TB symptoms as well as children <5 years were referred for clinical evaluation at the nearest PHC facility. Contacts’ socio-demographic and clinical characteristics, TB knowledge and perception about TB-related discrimination are described. Logistic regression analysis was used to investigate factors associated with non-attendance of clinical evaluation.

**Results:**

Out of the 259 participants, approximately three in every five (59.5%) were female. The median age was 20 (interquartile range: 8–41) years. While the large majority (87.3%) of adult contacts correctly described TB aetiology, almost three in every five (59.9%) thought that it was hereditary, and almost two-thirds (65.5%) believed that it could be cured by herbal medicine. About one-fifth (22.9%) of contacts believed that TB patients were subjected to discrimination. Two in every five (39.4%) contacts were referred for clinical evaluation of whom more than half (52.9%) did not attend the clinic. Non-attendance was significantly associated with inter alia male gender (AOR: 3.4; CI: 1.11–10.24), prior TB diagnosis (AOR: 5.6; CI: 1.13–27.90) and sharing of a bedroom with the index case (AOR: 3.4: CI: 1.07–10.59).

**Conclusion:**

The pilot study identified gaps in household contacts’ knowledge of TB. Further research on important individual, clinical and structural factors that can influence and should be considered in the planning, implementation and scale-up of household contact TB investigation is warranted.

## Background

For decades South Africa’s tuberculosis (TB) response has relied on a passive TB case finding approach, where individuals presenting to primary health care (PHC) facilities were screened and evaluated for TB. However, due to increasingly high numbers of undiagnosed individuals at community level, transmission rates [1, 2] as well as the risk for infection particularly among people living with human immunodeficiency virus (PLHIV), young children and close contacts of those with TB [3–5], escalated. As a result, active case finding strategies are recommended to supplement passive case finding. Moreover, active case finding strategies have been found to be highly cost-effective in South Africa, India and China [6].

Household contact TB investigation is one of a number of active case finding strategies recommended in high burden settings such as South Africa [7, 8]. Due to their close proximity to TB patients and resulting increased exposure to *bacilli*, household contacts are at higher risk for TB infection compared to the general population [4, 7, 8]. A study conducted in KwaZulu-Natal, South Africa, established that the TB incidence rate among household contacts of MDR-TB patients (1765 per 100,000 population) was 1.6 times higher than in the general population (1100 per 100,000 population) [4]. Additionally, a systematic review [9] reported an average TB prevalence of 3.1% among all contacts of TB patients in studies conducted in low and middle income countries. TB prevalence was particularly high among contacts ≤5 years (10.0%), household contacts of HIV co-infected patients (5.4%), household contacts of MDR-TB patients (3.4%), and household contacts of smear-positive patients (3.3%), and was lowest among TB patients’ casual contacts (2.2%). Consequently, it is thought that household contact TB investigation may facilitate early diagnosis and initiation of treatment, thereby reducing transmission in this population [8, 9].

Previous research in South African communities has established that targeting household contacts is more efficient than unselected community-based TB screening [10]. Contacts potentially exposed to an infectious TB patient may be accessed either through home visits by health workers [10, 11] or via special invitations to attend PHC facilities handed to contacts by the TB patients themselves [12]. In terms of outcomes, a higher yield of TB has been established by studies reporting home-based TB screening [10, 12] compared to research reporting on referral of contacts to PHC facilities for TB screening [12].

According to the South African TB management guidelines [5], contact investigation should be conducted promptly for all newly diagnosed pulmonary TB (PTB) patients. Following the launch of the revitalisation of PHC initiative in 2011, contact investigation/tracing is now incorporated into the household-level health promotion and disease prevention activities of ward-based outreach teams (WBOTs) [13–16]. Ideally, WBOTs comprise six community health workers (CHWs) supervised by a professional nurse and supported by environmental and health promotion practitioners [13, 16]. However, this is not a universal team make-up. Human resource limitations result in CHWs being supplanted by community caregivers (CCGs) with a lower training level. Another key challenge with the WBOT approach is the poor collection, storage and utilisation of data from households [15]. As such, it is unclear how regularly or systematically household contact investigation is undertaken by the teams.

This study targeted household contacts of newly diagnosed TB patients in a high burden district of the Free State, in line with WHO guidelines recommending that contacts of infectious TB patients including children < 5 years, smear-positive patients, HIV co-infected patients and MDR-TB patients should be prioritised for TB screening and evaluation [8]. The objective was to explore TB knowledge, perception of discrimination of TB patients, and factors influencing household contacts’ non-attendance of PHC facilities for TB clinical evaluation.

## Methods

### Design and setting

A pilot study was conducted in the Mangaung Metropolitan in the Free State Province in September to October 2016. In 2011, this metropolitan municipality or district had a population of 783,580 [17] and an average household size of approximately 3.1 persons [18]. In 2014, TB incidence was estimated at 686 per 100,000 population and in 2013, the cure rate among smear positive cases was estimated at 68.8% [17]. The high recorded TB patient numbers and concomitant poor treatment outcomes informed the purposeful selection of this district.

### Sampling, recruitment and data collection

The study population was defined as all undiagnosed household contacts of infectious TB patients who initiated treatment within 3 months prior to the field visit. A household contact was defined as a person who shared the same enclosed living space for one or more nights or spent frequent or extended periods during the day with the index case during the 3 months prior to commencement of the current treatment episode [8]. At every PHC facility at least one out of four types of index cases namely, children <5 years, smear-positive HIV negative cases (≥5 years), HIV co-infected cases (≥5 years) and MDR-TB cases, was selected. Household contacts of these purposefully selected index cases were then targeted for interviews and TB screening.

Arrangements to visit households were made through TB nurses attending to the index cases. The nurses informed the index cases (or caregivers of children <18 years) about the research and sought consent to provide their contact information to the researchers before the home visits. The nurses then referred the patients (or caregivers) to trained fieldworkers located in private rooms within the PHC facility for further information as well as to obtain consent for the household visits.

During the home visits, fieldworkers administered consent procedures before interviews were conducted with household contacts. Fieldworker-administered questionnaires were then completed with all household contacts present at the time of the visit. The questionnaire was developed based on instruments used in other studies [19–22] as well as the WHO guidelines recommending investigation of contacts of infectious TB patients [8].

Caregivers were interviewed on behalf of children <18 years. Information was collected on household contacts’ socio-demographic variables (e.g. gender, age, and relation to index case), TB knowledge, perception of TB-related discrimination, TB symptoms and contacts’ level of exposure to the index case. Additionally, fieldworkers used symptom-related questions — i.e. persistent cough, unintended weight loss, fever and night sweats [8] — to verbally screen household contacts for symptoms. On the one hand, a positive answer to any of the screening questions resulted in a referral for clinical evaluation at the nearest PHC facility. The contacts were referred to the nearest PHC facility for clinical investigation as the fieldworkers were not trained to collect sputum. On the other hand, if the contact answered ‘no’ to all screening questions, he/she was encouraged to present at the PHC facility as soon as such symptoms developed.

Household contacts were given evaluation forms with their identifying information, and reason/s for referral. At the PHC facility, the nurse conducted the necessary clinical evaluation and wrote the results on the evaluation form presented by the household contact. The fieldworkers collected the forms from the PHC facility within 2 weeks of the household contact being referred.

Two follow-up household visits as well as telephonic follow-up were undertaken by the fieldworkers to encourage clinical evaluation among contacts who had not presented to the PHC facility within the expected period. The outcome of the clinical evaluation was attached to the household contacts’ questionnaires before data were captured.

### Analysis

Data were processed and analysed using IBM SPSS statistics for windows, version 24. Frequency counts and percentages were used to describe participants’ socio-demographic and clinical information. Logistic regression analysis was employed to investigate the factors influencing non-attendance of PHC facilities for TB clinical evaluation. The independent variables included gender (male or female), age (as a continuous variable), whether the household contact had been diagnosed with TB before (yes or no), whether the household contact was coughing (yes or no), whether the household contact shared a bedroom with the TB index case (yes or no), time spent with index cases (12 hours or less or more than 12 hours per day), whether the contact had a swelling of the lymph nodes (yes or no), perception of discrimination against TB patients (yes or no), and preferred location for sputum collection (home or health facility).

### Study approval

The study was approved by the Ethics Committee of the Faculty of Health Sciences, University of the Free State (ECUFS 92/2013). Authorisation to conduct research at the PHC facilities was granted by the provincial Department of Health.

## Results

### Demographic information

Out of a total of 297 household contacts, 259 were enrolled into the study and 38 were excluded. Those excluded from the study included 10 who refused to be interviewed, and 28 who were not available for interviews due to work or travel commitments at the time of the field visit.

Out of the 259 participants, approximately three in every five were female (59.5%). The median age was 20 (interquartile range: 8–41) years. About one-quarter (25.5%) of the household contacts were children of the index cases. A substantial proportion (12.7%) of the household contacts reported a previous history of TB. Fever was the most frequent (16.6%) TB symptom reported. Just over one-third (36.6%) of household contacts shared a bedroom with the index case. The majority (75.3%) of household contacts had spent more than 12 hours per day with the index case prior to field visits. Two in every five (39.4%) household contacts were referred for clinical evaluation, of whom, more than half (52.9%) did not attend (Table 1).

**Table 1:** Characteristics of household contacts (*N* = 259)

Variable	n	%
Gender		
Female	154	59.5
Age in years		
< 5	36	13.9
5–15	73	28.2
16–24	33	12.7
25–34	34	13.1
35–44	25	9.7
45–54	19	7.3
55–64	18	6.9
≥ 65	21	8.1
Relationship with index case		
Spouse	21	8.1
Child	66	25.5
Other (e.g. sibling, aunt, uncle)	172	66.4
Had a history of TB	33	12.7
Presence of TB symptoms		
Cough	27	10.4
Haemoptysis	2	7.7
Unintentional weight loss	23	8.9
Fever	43	16.6
Night sweats	40	15.4
Had swollen lymph nodes	11	4.3
Tested for HIV	228	88.0
Slept in same bedroom as index case	94	36.3
Time spent with index case per day		
≤12 hours	64	24.7
>12 hours	195	75.3
Referred for clinical evaluation	102	39.4
Did not attend clinical evaluation^a^	54	52.9

^a^Out of those who were referred for clinical evaluation (*n* = 102)

### TB knowledge and perception of discrimination against TB patients

While an overwhelming majority (87.3%) of adult household contacts correctly described the aetiology of TB, almost three in every five (59.9%) misconstrued that TB is hereditary and just under two-thirds (65.5%) believed that herbal medicine can cure TB. The majority of household contacts could identify at-risk groups for TB including, children < 5 years (82.4%), PLHIV (84.5%), people with diabetes (67.9%), pregnant women (73.9%) and malnourished individuals (75.4%). Surprisingly, just over three-quarters (76.1%) and just under two-thirds (65.9%) incorrectly stated that leg pain and increased appetite were TB symptoms. The household contacts’ understanding of MDR-TB was moderate, with just over half agreeing that MDR-TB could be cured within 12 months (51.4%) and that MDR-TB patients’ household contacts should be screened for TB (57.7%) (Table 2). Regarding perception of TB discrimination, about one-fifth (22.9%) of household contacts believed that people with TB are discriminated against.

**Table 2:** Adult household contacts’ TB knowledge (*n* = 142)

Statement	Correct response n (%)
TB is caused by a bacterial infection (true)	124 (87.3)
TB is caused by living in an unhygienic environment (false)	130 (91.5)
TB is inherited from parents (false)	85 (59.9)
Smoking tobacco increases susceptibility to TB (true)	128 (90.1)
Herbal medicine can cure TB (false)	93 (65.5)
Risk for TB is increased among:	
Children under five years (true)	117 (82.4)
PLHIV (true)	120 (84.5)
People with diabetes (true)	96 (67.6)
Pregnant women (true)	105 (73.9)
Malnourished people (true)	104 (73.2)
TB can severely affect a person’s health (true)	107 (75.4)
Symptoms commonly associated with TB infection:	
Cough (true)	121 (85.2)
Fatigue (true)	109 (76.8)
Leg pain (false)	108 (23.9)
Weight loss (true)	132 (93.0)
Night sweats (true)	126 (88.7)
Increased appetite (false)	47 (33.1)
Coughing up blood (true)	117 (82.4)
Chest pain (true)	122 (85.9)
Dizziness (false)	121 (85.2)
Fever (true)	117 (82.4)
Difficulty breathing (true)	117 (82.4)
MDR-TB can be cured within 12 months (true)	73 (51.4)
Household contacts of MDR-TB patients should undergo screening (true)	82 (57.7)

### Factors associated with non-attendance of PHC facilities for clinical evaluation following referral

A binomial logistic regression was performed to ascertain the factors influencing household contacts’ non-attendance of TB clinical evaluation. The assumption of linearity of continuous variables was assessed using the Box Tidwell procedure. Based on this assessment, all continuous variables were linearly related to the logit of the dependent variable. Three outliers, one high leverage value and one influential point were identified. Since these unusual points were not due to data entry errors, they were maintained in the analysis. A test of the full model against a constant only model was statistically significant (chi-square = 19.498, *p* < 0.034, df = 10), indicating that the predictors as a set, reliably distinguished between attendance and non-attendance of PHC facilities for TB clinical evaluation. The model explained 28.9% (Nagelkerke’s R^2^) of the variance of non-attendance of clinical evaluation. The overall prediction success was 70.7% with specificity of 50.0% and sensitivity of 82.7%. The positive predictive value was 74.1% and the negative predictive value was 62.5%.

After controlling for all variables in the model, gender, age, prior TB diagnosis, whether household contacts shared a bedroom with the index case, and whether they preferred sputum (for microscopy) to be collected at home or at the PHC facility were statistically significantly associated with non-attendance of PHC facilities for TB clinical evaluation. Compared to their female counterparts, male household contacts were 3.4 (CI: 1.11–10.24) times more likely avoid clinical evaluation. Also, the odds for non-attendance of clinical evaluation increased by 1.03 (CI: 1.003–1.057) times with every unit increase in age. Non-attendance of clinical evaluation for TB was 5.6 (CI: 1.13–27.90) times more likely among household contacts with a prior history of TB compared to those without and 3.4 (CI: 1.07–10.59) times more likely among those who shared a bedroom with an index case compared with those who did not. Results further showed a 16-fold (CI: 1.17–226.84) higher likelihood for non-attendance of clinical evaluation among household contacts who preferred sputum collection to be done at their homes relative to those who preferred this to be done at the PHC facility (Table 3).

**Table 3:** Factors associated with non-attendance of PHC facilities for TB clinical evaluation

Variable	Non-attendance of clinical evaluation (*n* = 54)	Adjusted odds ratio	95% confidence interval	*p*-value
	n (%)			
Sex				
Female (ref)	32 (59.3)	1		
Male	22 (40.7)	3.4	1.11–10.24	0.032
Age (mean; standard deviation)	29.4 (22.8)	1.03	1.003–1.057	0.029
Has been diagnosed with TB before				
No (ref)	42 (77.8)	1	1	
Yes	12 (22.2)	5.6	1.13–27.90	0.035
Is coughing				
No (ref)	38 (70.4)	1		
Yes	16 (29.6)	1.7	0.48–5.85	0.425
Time spent with index case				
Only night/day (ref)	46 (85.2)	1		
All the time	8 (14.8)	1.0	0.23–4.00	0.961
Shares bedroom with index case				
No (ref)	28 (51.9)	1		
Yes	26 (48.1)	3.4	1.07–10.59	0.038
Has had swelling of lymph nodes				
No (ref)	50 (92.6)	1		
Yes	4 (7.4)	0.2	0.02–1.34	0.094
Perceives discrimination against TB patients (*n* = 52)				
No (ref)	42 (80.8)	1		
Yes	10 (19.2)	1.1	0.27–4.11	0.941
Preferred location for sputum collection				
Health facility (ref)	46 (85.2)	1		
Home	8 (14.8)	16	1.17–226.84	0.038
TB knowledge (mean, standard deviation)	8.5 (2.0)	0.9	0.71–1.18	0.543

In terms of reasons for non-attendance of PHC facilities for clinical evaluation, not everyone that avoided clinical evaluation provided a reason. The most cited reason for non-attendance of PHC facilities for clinical evaluation was difficulty to get time off other duties such as work and school (44.4%). Some household contacts also anticipated that they would not be able to produce sputum for assessment (15.6%). Lack of transportation to PHC facilities (11.1%), travel commitments (11.1%), long queues (8.9%) and perception of unhelpful staff (8.9%) at PHC facilities were also mentioned. Thereupon, actually becoming ill [with TB] (37.5%), sensitisation about TB screening and evaluation (25.0%), shorter waiting times at PHC facilities (25.0%) and being encouraged by someone, would motivate household contacts to attend clinical evaluation (Table 4).

**Table 4:** Household contacts’ reasons for non-attendance of PHC facilities and perceived facilitators of clinical evaluation for TB

Reason for non-attendance of clinical evaluation	*n* = 45	%
Difficulty to get to get time off other duties (e.g. work, school)	20	44.4
Perceived inability to produce sputum for assessment	7	15.6
Lack of transport	5	11.1
Travel commitments	5	11.1
Long queues at clinic	4	8.9
PHC facility staff perceived as unhelpful	4	8.9
Facilitators of clinical evaluation	*n* = 8	%
Becoming ill (with TB)	3	37.5
Sensitisation about TB screening and evaluation	2	25.0
Shorter waiting times at clinics	2	25.0
Being encouraged by someone	1	12.5

## Discussion

This study investigated TB knowledge, perception of discrimination of TB patients, and factors influencing household contacts’ non-attendance of PHC facilities for TB clinical evaluation. In line with a previous study conducted among TB patients and their household contacts in Vietnam [22], most participants in this study were knowledgeable about the aetiology of TB, risk groups for and symptoms of TB. However, the household contacts’ understanding of MDR-TB was moderate at best, suggesting a need to improve health education about MDR-TB among this risk group. Additionally, a substantial proportion of household contacts said that TB is hereditary, with some indicating that it can be cured with herbal medicines. These incorrect beliefs about TB could be linked to traditional influences as previous research has reported that some communities in South Africa have a high regard for traditional healers and tend to believe that they can treat TB with herbs [23–25]. If left unaddressed, such incorrect beliefs about TB could have negative implications on household contacts’ health seeking behaviour, as well as general TB control efforts.

About one-fifth of household contacts believed that people with TB are discriminated against. While the odds were not statistically significant, those who perceived TB patients to be discriminated against were also more likely to avoid TB clinical evaluation at PHC facilities. It is important to address extant risks such as discrimination if TB case finding is to be successful [22, 26, 27]. In Nigeria, a health education intervention was used to increase TB patients’ awareness about contact screening. Patients were also skilled to avert negative behaviours and attitudes towards TB screening and to encourage their contacts to undertake TB screening, consequently improving the rate of case detection [26].

Male gender and advancing age significantly influenced non-attendance of clinical evaluation. The finding on male household contacts’ higher likelihood for non-attendance of clinical evaluation for TB confirms previous research on contact tracing in the Western Cape Province, South Africa. The study found that males were less likely to visit PHC facilities for TB screening when requested, purporting that men visiting clinics are not masculine [12]. In another active case finding study in Gauteng Province, South Africa, portable gazebos were erected in minibus parking bays and taxi drivers (all male) were invited to screen for TB. While this approach brought the TB service closer to the drivers and subsequently reduced waiting time, uptake of TB screening among the taxi drivers remained poor [28]. Special programme efforts such as after-hour services may be necessary to encourage males to partake in TB control, especially TB evaluation.

The observed increased likelihood of non-attendance of clinical evaluation with every unit increase in age confirms findings of a study among TB patients in Ethiopia where both diagnostic and treatment delay were associated with older age [29]. However, the current finding contradicts the findings of a study among PHC facility attendees in Gauteng Province, South Africa, where older people were more likely to seek TB services compared to younger ones [30].

In terms of clinical factors, persons with previous TB diagnosis were almost six times more likely to miss clinic evaluation compared to their counterparts who had never had TB. This group of people had already been subjected to previous prolonged and sometimes complex TB treatment, possibly evoking negative emotions towards TB programme activities. Although this was not measured, they may also have been subjected to stigmatisation/discrimination. As research has established a high prevalence of TB among people who were previously treated for TB [31], TB programmes need to prioritise this group when designing contact screening and evaluation programmes.

The household contacts who reported sharing bedrooms with TB index cases were more likely to miss clinical evaluation. As indicated in a case-control study in The Gambia, TB was associated with increasing household size, with at least two individuals sharing bedrooms [32]. In a Vietnam study, cases were less likely than controls to perceive that sharing bedrooms with TB patients increased the likelihood for TB transmission and were also less likely to attend TB screening [22]. Household contacts in the current study may have avoided clinical evaluation due to suspicion that they might already be infected. This points to the need for the TB programme to strengthen the notion that TB is curable and that early seeking of diagnosis and treatment is useful.

The finding that household contacts preferring sputum collection at their homes relative to PHC facilities were less likely to attend PHC facilities for clinical evaluation suggests the need to strengthen home-based TB services. As mentioned before, in this study fieldworkers verbally screened household contacts for symptoms. Those who were symptomatic as well as children <5 years were referred to the PHC facilities for sputum collection and clinical evaluation. Due to resource constraints only two follow-up household visits were undertaken to reach all contacts and to screen incident cases in this study. A previous study conducted in the Western Cape Province, South Africa [12] relied on paper-slip invitations issued through index TB patients to their close contacts requesting them to visit their nearest PHC facility for TB investigations, and only 26% of the participants honoured these invitations. Clearly, the referral approach is limited in ensuring that household contacts visit PHC facility for clinical evaluation. Thus, TB programmes should consider alternative strategies to ensure clinical evaluation such as use of specialised community-based lay workers for contact tracing as this may accommodate repeat visits as well as visits outside of working hours [33].

Most household contacts in the current study missed clinical evaluation for personal reasons including lack of time, perceived inability to produce sputum samples, lack of transport to clinics and other travel commitments. Service-related deterrents to clinical evaluation were long queues at PHC facilities and perceptions of staff being unhelpful. Similar explanations emerged from a study conducted in Vietnam among patients and their household contacts [22] and should therefore be taken into consideration during scale-up efforts.

Limitations of the pilot study include that it was restricted to a purposively selected sample of household contacts based on the WHO’s four categories of index cases (at least one per clinic) which limits the generalisability of the results. While the small sample allowed for exploration of the reasons for non-attendance of clinical evaluation by household contacts, it ruled out the possibility of assessing the degree of exposure between them. Also, because field visits were conducted within working hours only, some household contacts including those who were at work – particularly males – or at school, were not reached. Additionally, although contact tracing is one of several functions of WBOTs [13, 16], it was particularly challenging in this study to find outreach teams to assist with home-based sputum collection or follow-up of contacts who had been screened and referred to PHC facilities for clinical evaluation. Indeed, previous research has shown that outreach based teams’ efficiency is often compromised by resource constraints, staff shortage and work overload [15, 16].

## Conclusion

This pilot study identified gaps regarding household contacts’ knowledge of TB. The study further found that non-attendance of clinical evaluation was high and was significantly influenced by male gender, advancing age, prior TB diagnosis, sharing of a bedroom with the index case and preference for sputum collection at home rather than at the PHC facility. It is recommended that the highlighted individual, clinical and structural factors that can influence household contact TB investigation scale-up should be further subjected to more representative research.

## Abbreviations

MDR-TB: Multidrug-resistant tuberculosis
PHC: Primary health care
PLHIV: People living with human immunodeficiency virus
PTB: Pulmonary tuberculosis
TB: Tuberculosis
WBOT: Ward-Based Outreach Team
WHO: World Health Organization
